# Recommendations for the Quality Management of Patient-Generated Health Data in Remote Patient Monitoring: Mixed Methods Study

**DOI:** 10.2196/35917

**Published:** 2023-02-24

**Authors:** Robab Abdolkhani, Kathleen Gray, Ann Borda, Ruth DeSouza

**Affiliations:** 1 Centre for Digital Transformation of Health The University of Melbourne Melbourne Australia; 2 Department of General Practice Melbourne Medical School The University of Melbourne Melbourne Australia; 3 Faculty of Medicine, Dentistry and Health Sciences The University of Melbourne Melbourne Australia; 4 School of Art Royal Melbourne Institue of Technology University Melbourne Australia

**Keywords:** data quality management, patient-generated health data, remote patient monitoring, wearable electronic devices, remote sensing technology, telemedicine, big data

## Abstract

**Background:**

Patient-generated health data (PGHD) collected from innovative wearables are enabling health care to shift to outside clinical settings through remote patient monitoring (RPM) initiatives. However, PGHD are collected continuously under the patient’s responsibility in rapidly changing circumstances during the patient’s daily life. This poses risks to the quality of PGHD and, in turn, reduces their trustworthiness and fitness for use in clinical practice.

**Objective:**

Using a sociotechnical health informatics lens, we developed a data quality management (DQM) guideline for PGHD captured from wearable devices used in RPM with the objective of investigating how DQM principles can be applied to ensure that PGHD can reliably inform clinical decision-making in RPM.

**Methods:**

First, clinicians, health information specialists, and MedTech industry representatives with experience in RPM were interviewed to identify DQM challenges. Second, these stakeholder groups were joined by patient representatives in a workshop to co-design potential solutions to meet the expectations of all the stakeholders. Third, the findings, along with the literature and policy review results, were interpreted to construct a guideline. Finally, we validated the guideline through a Delphi survey of international health informatics and health information management experts.

**Results:**

The guideline constructed in this study comprised 19 recommendations across 7 aspects of DQM. It explicitly addressed the needs of patients and clinicians but implied that there must be collaboration among all stakeholders to meet these needs.

**Conclusions:**

The increasing proliferation of PGHD from wearables in RPM requires a systematic approach to DQM so that these data can be reliably used in clinical care. The developed guideline is an important next step toward safe RPM.

## Introduction

### Remote Patient Monitoring

The use of remote patient monitoring (RPM) solutions and production of patient-generated health data (PGHD) to enable continuous monitoring of patients outside clinical settings are increasing with the growing availability of health wearable devices and the connected mobile apps and web portals [1]. The COVID-19 pandemic has accelerated the use of RPM to monitor mild cases of the disease remotely, given the limited capacity of acute care facilities [2].

As the pandemic is not yet over, RPM will likely contribute more to health care delivery owing to the availability of various affordable technologies and the need for remote treatment and monitoring. However, despite the urgent need and rapid implementation and use of RPM, investigation on how quality PGHD can best be collected and managed to lead to accurate decision-making is still lacking.

### Ensuring the Quality of PGHD

Patients may collect some data as instructed by clinicians, mainly from medical wearables. Patients may also collect data, on their own accord or on advice from clinicians, from consumer wearables. There are fundamental similarities between the data collected upon patient initiation and those collected upon clinician initiation, whether from consumer or medical wearables, that erode the regulators’ distinctions: the data are generated outside the controlled environment of the clinic; the data collection is the responsibility of the individual wearer; and the data are shared electronically with parties who operate outside a controlled clinical setting, namely wearable companies. Thus, RPM data collected from wearables, whether upon patient initiation or upon clinician initiation, are covered by the broad concept of PGHD.

Outside the clinic, consumer and medical wearable technologies used in RPM capture a large amount of data continuously in rapidly changing circumstances during a patient’s daily life under the patient’s or caregiver’s supervision [3]. The wearable platform includes sensors that capture data automatically and a mobile app and web portal where the person enters data manually. Inside the clinic, RPM solutions are not integrated well into patient records or clinical workflows, and various digital health devices and platforms are used for different RPM purposes [4]. The quality of PGHD collected from disparate devices is compromised by various technical, behavioral, or operational issues that occur during data capture by the patient or caregiver, during the transmission of the data from the patient to the clinician, and during the clinician’s review of the data for decision-making [5].

Health data quality plays a vital role in health care systems. Clinicians need to trust the available data to make accurate decisions and provide efficient and timely care for their patients. Data are of good quality when they are fit for their intended use [6], that is, when they are accurate, accessible, consistent, complete, interpretable, timely, relevant, and compliant with the standards defined by health care organizations [7]. Any quality issue with data can affect patient safety, the reimbursement of health services, and the quality of clinical outcomes and other aspects of health care delivery [8].

Data quality management (DQM) refers to the processes of ensuring data quality when data are collected, stored, analyzed, reviewed, and used in clinical decision-making [9]. The core outcome of DQM is establishing the fitness of data for its intended use. National and international health care and health information–related organizations provide guidelines for the quality management of patient data that are generated within clinical settings [8-12]. However, in RPM, data are collected outside the clinical setting, and different stakeholders are involved at different stages of PGHD management both outside and inside the health care settings.

This paper describes the DQM recommendations provided to ensure that data from wearables are fit for use in clinical care.

## Methods

### Overview

Recommendations for the quality management of PGHD arose from a mixed methods study on the quality management of PGHD from wearables and were constructed following a guideline development convention in health care [13-18] through the stages listed in Textbox 1.

Most of the data collection in this research was done before the COVID-19 pandemic. However, the rapid deployment of RPM during the pandemic emphasizes the need for guidelines, such as the one constructed in this study, to improve the use of RPM initiatives and efficiently integrate them into the routine care.

**Stages involved the construction of the recommendations.**

**Figure 1:**
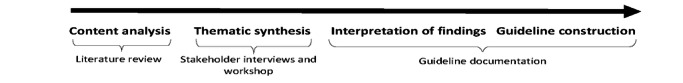
Continuum of integrating multiple qualitative findings to create new evidence.

- Evidence reviews: a comprehensive literature review focused on original research within a 10-year time frame that discussed the barriers to and concerns of using patient-generated health data (PGHD) in clinical practice was conducted and published previously [19].
- Stakeholder involvement: in-depth interviews were conducted with PGHD stakeholders directly involved in the remote monitoring of patients with chronic diseases, including those with diabetes, those with cardiac arrythmia, and those sleep disorders, in primary care, secondary care, and tertiary care settings to identify the challenges related to the quality management of PGHD. The interview participants were from Australia, the United Kingdom, and the United States. The interview results were published previously [5]. Then, a participatory workshop was held in Australia with stakeholders, including patients, clinicians, health information professionals, wearable developer companies, PGHD integration service providers and remote patient monitoring (RPM) consultants, to discuss the identified challenges and address potential solutions and stakeholders’ needs and expectations. The results of this study were published elsewhere [20].
- Documentation of recommendations: we used the approach of integrating multiple types of qualitative evidence to produce new knowledge [21], as shown in Figure 1, to construct a set of recommendations that cover all aspects of the quality management of PGHD during data flow from the patient to the clinician. We synthesized the findings of the aforementioned 2 stages along with supporting evidence from an updated review of scientific literature and new policies related to PGHD. The interpretation stage aimed to draw connections between the data points, themes, and findings. Then, the construction stage expressed new meaning and uncovered ways of understanding the realities regarding the research topic for the stakeholders. It acknowledged the importance of synthesized and interpreted elements in terms of stakeholders, context, and influencing issues. Moving from evidence to recommendations, the construction stage sought to examine how the synthesized findings related to the broader context in the past, present, and future. Details of the findings and the emerged themes applied in the construction of the guideline are provided in Multimedia Appendix 1. This step produced a guideline containing 19 separate recommendations. One of the researchers interpreted the findings and constructed the guideline, and then the guideline content was reviewed separately by 3 researchers and discussed in multiple meetings.
- Validation of recommendations: a 1-round Delphi method [22] using a web-based survey and 5-point Likert scale was adopted, 14 Australian international health informatics and health information management experts participated in the survey. The interview and workshop studies involved several groups of PGHD stakeholders, who shared their experience and perspectives related to the quality management of PGHD. However, except for health information professionals, the other stakeholders were experts in their own clinical or technical field but not in managing and governing patient health data. The data quality management (DQM) elements in the guideline still required validation by experts with a high-level understanding of and experience with health information management and health informatics principles and practices. These experts were purposefully selected based on their professional reputation and their known interest in PGHD. None of them had been involved in the previous studies of this project, so they could form an independent view of the resulting recommendations. The survey had 19 items in total, representing the 19 recommendations in the guideline. It used a 5-point Likert scale (not important, slightly important, moderately important, important, and very important) to capture the participants’ expert opinions about the extent to which each DQM recommendation is potentially important in contributing to the safety and the quality of RPM. Each survey item also included a free-text comment option so that the participants could further explain their response. Consensus on each recommendation for each DQM aspect of PGHD was deemed to be achieved by having 60% of votes fall within 2 adjacent categories of the 5-point scale. A method to group the responses for analysis was determined: if the participants reached at least an aggregated 60% agreement that a recommendation is “important” or “very important,” it was deemed to have been rated as essential; if the participants reached at least an aggregated 60% agreement that a recommendation is “slightly important” or “moderately important,” it was deemed to have been rated as desirable; and if the participants reached at least an aggregated 60% agreement that a recommendation is “not important,” it was deemed to have been rated as unnecessary.

### Ethics Approval

The stakeholder involvement studies received approval from the Human Ethics Advisory Group at the Department of General Practice at the University of Melbourne [5,20]. The ethics approval number for the validation study from the same group is 1955682.1.

## Results

### Recommendations and Key Themes

The ensuing guideline encompasses 19 recommendations. These recommendations were grouped according to 7 overarching DQM aspects. Table 1 lists these 7 aspects; their adapted definition for this research; and the key themes identified from the literature review, interviews, and workshop studies. The sociotechnical issues to be considered in relation to each DQM aspect have been discussed in the corresponding recommendations. Through this style of presentation, PGHD stakeholders can understand what actions they and others need to take to collect, manage, and use trustworthy PGHD in RPM.

**Table 1:** Data quality management (DQM) recommendations for patient-generated health data (PGHD) in remote patient monitoring.

DQM aspects and key themes		Recommendations
**PGHD accessibility: authorized users of PGHD access t** **hem** **across all data management stages**		
	Patients’ and clinicians’ access to PGHD	Both raw and processed PGHD from wearables should be accessible to the patient and clinician.
	Patients’ and clinicians’ awareness of PGHD access by others	A mechanism should be available to the patient and clinician to set up notice recurrence on where, when, how, and by whom PGHD from wearables are accessed.
	Patients’ consent to PGHD access by different clinicians	A mechanism should be available to the patient to change permissions for clinicians to access PGHD from wearables.
**PGHD accuracy: error-free data**		
	Automatic and manual PGHD collection	PGHD should be collected automatically by the wearable device, with as little as possible manual intervention.
	PGHD annotation	Annotation function for manually and automatically entered PGHD should be available to the patient and clinician in order to comment on inaccurate data.
	Wearable calibration	The wearable should be calibrated automatically as required by the clinical standard of care of diseases.
**PGHD completeness: no PGHD are missing**		
	No active data collection	A protocol should be available to the patient and clinician that defines PGHD “downtime,” that is, the time range during which it is acceptable if the wearable is not collecting data.
	Resuming PGHD collection after downtime	A protocol should be available to the patient and clinician for resuming PGHD collection when the acceptable downtime period is exceeded.
	Context for incomplete PGHD	Annotation function should be available to the patient in order to provide context for any period of missing PGHD.
**PGHD consistency: data convey the same meaning no matter whether they are collected from one or different brands of wearables**		
	PGHD definitions and formats	PGHD from wearables should be collected based on clinically accepted and structured data definitions and standard formats.
	PGHD integration with electronic medical records	PGHD from wearables should be integrated into the patient’s clinical care record.
	PGHD exchange within and outside care settings	PGHD from wearables should be consistently exchanged inside and between clinical settings.
**PGHD interoperability: data presentation highlights the key message that is understood by PGHD stakeholders**		
	PGHD contextualization	PGHD from wearables should be accompanied by contextual data that are clinically important to patient management.
	Dynamic and static PGHD visualization	Dynamic visual representation as well as a static snapshot (such as in PDF format) of PGHD from wearables should be available to the patient and clinician.
	The patient’s understanding of PGHD	Alerts should be sent to the patient during PGHD collection by the wearable when data are outside the acceptable range, accompanied by clinical advice on action to take.
**PGHD relevancy: data are pertinent to the standard of care for the condition being monitored**		
	PGHD relevancy to the standards of care	There should be a shared understanding between the patient and clinician of relevant data for the disease based on the standards of care and make sure that all the relevant data are collected.
**PGHD timeliness: availability of up-to-date PGHD for patients and clinicians when needed**		
	PGHD availability to patients when needed	PGHD from wearables should be available to the patient within a timeframe (continuously to periodically) according to the standards of care of diseases.
	PGHD availability to clinicians when needed	PGHD from wearables should be available to the clinician within a timeframe (continuously to periodically) according to the standards of care of diseases.
	Time frame for PGHD sharing between patients and clinicians	A timeframe for sharing PGHD from wearables should be available to the patient and clinician.

### PGHD Accessibility

PGHD accessibility was characterized by data access methods, privacy protection, and data ownership issues to be explored in RPM.

#### Recommendation 1: Both Raw and Processed PGHD From Wearables Should Be Accessible to the Patient and Clinician

The extent to which patients and clinicians currently have access to all the recorded PGHD is questionable. PGHD accessibility largely depends on who owns the data to have complete access to them. PGHD have not yet been fully incorporated into clinical workflows; therefore, these data are neither controlled nor owned by health care organizations. Rather, the raw and processed PGHD from each wearable platform are accessed and controlled by the device company outside the health care setting.

Access to raw and processed PGHD during data collection may increase patients’ awareness of their health status and whether they are required to take action or change their behavior and improve self-care. Now, the trend in wearable design is shifting toward data visibility to patients [23,24]. Nevertheless, clinicians may intentionally disable the access of raw data to patients during data collection, as it would lead to patient behavior change that might conflict with the purpose of the RPM program. The ability to access all raw and processed PGHD could also be limited by wearable companies. Medical device manufacturers should share comprehensive and contemporary health information with patients upon request [25]. Therefore, patients are within their rights to request health information that is captured, stored, and analyzed by and retrieved from a legally marketed medical device. Different policies suggest that wearable developers, regardless of the wearable type, should provide patients complimentary access to PGHD [26-28].

Considering these policies, PGHD ownership has not yet been defined clearly enough to determine who owns part or all of the data, affecting patients’ access to PGHD [29,30].

In terms of clinicians’ access to raw and processed PGHD, the necessity to access all the collected raw and processed data depends on which data are needed for decision-making. Our findings showed that it would be difficult for a clinician to find the log-in details of a patient’s wearable portal if the patient has changed their portal account information or the device without informing the clinician. Clinicians’ access to PGHD might also be prevented by patients, which might reveal that they have not followed their care plans [31].

Collecting various types of PGHD from different wearables outside the clinical environment means that data are stored across different platforms. Ideally, PGHD should be accessible to the people who collect them, and access methods should be transparent. The purpose of giving patients and clinicians access to PGHD is to enable them to have a clear picture of the former’s health status.

#### Recommendation 2: A Mechanism Should Be Available to the Patient and Clinician to Set Up Notice Recurrence on Where, When, How, and by Whom PGHD From Wearables Are Accessed

It is important that patients and clinicians be aware of who else has access to PGHD during data management from outside the health care setting to inside it. Patients and clinicians in this project had little understanding of how and by whom PGHD are accessed during data management stages in RPM [32]. Clinicians placed responsibility on the wearable developer for informing patients about who can access their data. Also, the installation terms and conditions of a large number of health wearables’ apps indicate that the wearable developers are the owners of PGHD and have authority to grant data access to others [26]. A review study of 4 known wearable products showed that the privacy policy of only 1 platform asserted PGHD as users’ sole and exclusive property [33]. However, these companies’ statements were not accompanied by strategies to support patients’ awareness of the accessibility of their data to others. Patients should be informed about what PGHD are collected and accessed, including possible lawful access by third parties; whether these data are identifiable or depersonalized; and how they are accessible for clinical decision-making [27,28,34,35]. Patients need transparency about PGHD access not only before data collection but also throughout all the PGHD management stages. Not knowing who has access to their data can deter or inhibit PGHD collection [23].

Initiatives such as the privacy notice checklist developed by the US Office of National Coordinator for Health Information Technology are to be used by wearable developer companies to disclose their privacy and security policies to patients and inform them about what happens to their PGHD once they purchase and use the device [36]. However, this notice appears to be more applicable to wearables for self-management than to those for RPM. In addition, one-off use of the privacy notice checklist cannot ensure the notification of all PGHD accesses during all data management stages. For example, PGHD might be transferred from the patient to the clinician through communication networks that might be hacked. Many RPM programs lack robust cybersecurity mechanisms [37].

Using PGHD in clinical practice means that clinicians might also need to be notified about PGHD flow to be able to track patient monitoring instructions from other clinicians if necessary. Patients and clinicians should be able to set up notification recurrence of PGHD access based on their preference.

#### Recommendation 3: A Mechanism Should Be Available to the Patient to Change Permissions That Clinicians Have to Access PGHD From Wearables

The patients’ and clinicians’ awareness of circumstances under which PGHD are accessed does not give patients the authority to consent to PGHD access by others.

It is unclear to PGHD stakeholders how patient’s consent to PGHD access should look [38]. Patients in the RPM of our 3 use cases, diabetes, cardiac arrhythmia, and sleep disorders, sign a consent form at the beginning of the program. However, the continuous nature of data collection and access in RPM might require constant PGHD access authorization when different clinicians need to access the data for different purposes of patient care [29,39]. There are concerns that the clinicians may access PGHD at a stage where the data have not been granted access to by the patient. This might not be ethical even if done to benefit the patient [40].

Patients themselves may have little awareness of PGHD consent, and their attention may be confined to the terms and conditions statement before pressing the consent button for installing the wearable components. However, the wearable developers’ privacy policies and terms of service are often difficult to read and understand [41,42].

Appropriate consent management mechanisms enable patients to manage their consent preferences. Nevertheless, there is not yet a well-established consent mechanism for continuous data collection and use [28]. As various types of PGHD might be collected through different wearable platforms, sensitive data might be released when using one consent at the beginning of the RPM program. For example, patients might not want to provide details about their behaviors or lifestyles to clinicians in a certain time frame if it would lead to judgment or being shamed for perceived unhealthy choices during data collection. Thus, a process of dynamic consent might be more feasible to give patients control over the level of access to their data for different purposes and the choice of whether these data are anonymized or identifiable [43,44]. It could provide more personalized approaches and improve the continuous patient-clinician communication. Also, it gives patients the ability to understand and decide to what extent they are willing to share their data. Moreover, defining different levels of permission enables patients to review consent over a period to update or withdraw data at any time without affecting previously collected data [28].

#### Validation Results

Each of these 3 recommendations about PGHD accessibility was rated as “essential” to the to the safety and quality of care in RPM (reached an aggregated 60% agreement as being important to very important).

### PGHD Accuracy

PGHD accuracy is compromised by a patient’s errors or other error sources during data management, as well as uncontrolled possibilities for data revision.

This aspect depends on the technical features of the wearable and its components and the behaviors of the patient or caregiver at the point of data collection. Clinicians’ trust of PGHD accuracy is significantly impacted by the differentiation between medical grade and consumer wearables. Clinicians trust the level of accuracy in PGHD captured by medical wearables owing to their preassessment and approval from regulatory bodies. Our findings showed that consumer wearables were not used in RPM because of not being regulated for clinical use. However, even a medical wearable may not work accurately in some instances, as identified in our interviews and workshop studies. Moreover, a study showed that the inaccuracy of continuous glucose monitoring (CGM) wearables was the most critical impediment (53%) to the use of these devices by diabetic adults [45]. Nevertheless, as the wearables collect data longitudinally, clinicians may trust the overall trends rather than doubting whether a single data point was captured correctly.

#### Recommendation 4: PGHD Should Be Collected Automatically by the Wearable Device, With as Little as Possible Manual Intervention

The way PGHD are collected can pose risks for data accuracy. Automated sensing via algorithms embedded into wearables can provide persistent collection and analysis, providing a comprehensive picture of a patient’s status over time. Automation can lower the tracking burden, improve PGHD accuracy, and accelerate data filtering for timely access [39]. For a patient with low digital health literacy, automated data collection can reduce the level of disengagement with the device.

In addition to automatic data collection, some wearables require types of PGHD such as meal, activity, and mood data to be entered manually in the wearable platform on a daily basis. This can place a burden on patients and result in inaccurate and inconsistent recordings [46]. Yet, there has been no innovation to change the manual collection of these types of PGHD into a seamless automatic process; however, the extent of engagement in manual PGHD collection and documentation might depend on the patients’ level of understanding of the data and the message that PGHD could convey to the patients [47]. From clinicians’ perspectives [48,49], automated data collection and transmission to the associated app is a more accurate mechanism to evaluate peak flow variability than a patient’s difficult and time-consuming manual calculations.

Some PGHD types that patients were required to record manually—such as activity data in the remote monitoring of patients with diabetes—could be automatically captured via consumer wearables. Synchronization shortages between different types of medical and consumer wearables and a lack of adoption of consumer wearables in RPM are barriers to increasing automation. Although it might not yet be possible for some data elements to be captured automatically, there could be strategies to limit free-text entries. For example, wearable developers can reduce the possibility of errors in manual data entry in the associated apps by requiring the user to choose from a list of options instead of entering free-text [50].

However, having all PGHD collected automatically may lead to less control by patients over their health status and reduce their engagement in their self-care [51]. In addition, behavioral factors such as improper application of a sensor on the body or changing the device settings can have adverse impacts on PGHD accuracy. Automation can provide more accurate data if it does not negatively impact patients’ engagement in self-care.

#### Recommendation 5: Annotation Function for Manually and Automatically Entered PGHD Should Be Available to the Patient and Clinician in Order to Comment on Inaccurate Data

Whether PGHD are collected automatically or manually, the ability to annotate them during data collection is a critical contribution to their accuracy [31]. In addition to the annotation of manual entries, the annotation of automatically collected data can help patients prevent errors in them and mark questionable data to discuss with clinicians [52].

The rapidly changing environment surrounding the patients may contribute to inaccuracies in manual and automatic captures [53]. Sometimes, the wearable works inappropriately or the patient makes mistakes in wearing the device or entering data; however, the feature of annotating both data collected manually and those collected automatically is not designed in many wearable platforms and is often overlooked in the testing of wearables for use in RPM [47].

According to our findings, patients can add notes on inaccuracies only through their diaries to discuss them with clinicians during the clinical consultations. However, the annotation feature could be embedded in the wearable design to reflect on data inaccuracy in real time instead of writing a diary note that might be forgotten. Patients could be notified of the incorrect values to annotate data or redo data collection instead of sending incorrect data to the clinician [30]. The wearable developers could also enable passive data annotation upon patients’ request [28]. Nonetheless, it is uncertain, if the patients themselves do not notice the errors, how they could annotate PGHD given that wearables often lack feedback mechanisms to alert the wearer about inaccuracies [54].

There is a concern that patients may use this functionality to override real actions. Therefore, the patients and caregivers need to be educated on PGHD annotation and build trust upon this functionality to enhance patient-clinician interaction and shared decision-making [46,55].

Clinicians should also be able to annotate the processed PGHD to understand data collection barriers and provide more efficient personalized care plans [29]. However, as the processed PGHD are usually represented as static snapshots to the clinician, it would be difficult for a clinician to annotate the reports and highlight the problematic areas of PGHD [56,57].

#### Recommendation 6: The Wearable Should Be Calibrated Automatically as Required by the Clinical Standard of Care of Diseases

Both medical and consumer wearables may collect inaccurate data. Therefore, it is important to ensure that wearables are calibrated to guarantee accurate sensing [30,39,58].

Some wearables need one-off calibration by the clinicians before initiating remote monitoring, whereas for other types such as CGM devices, the patient should frequently calibrate the device via a glucometer to ensure PGHD accuracy. Nonetheless, a patient’s responsibility in terms of how often and when they should calibrate the wearable device in RPM is often unclear [31]. The need to calibrate wearables not only is a burden on patients or their caregivers but also increases the likelihood of inaccuracies. Patients should be taught the importance of calibration and when it should be done. For example, from a clinician’s point of view, the 12-hour calibration for CGM wearables prescribed by most wearable developer companies [59] is not clinically acceptable; rather, calibration should be done 3 times a day when the blood glucose is not rapidly changing. Similarly, the best time to calibrate the device is when the glucose level is stable [60]. A study showed that nearly half of the participants reported calibrating CGM at more intervals than recommended by the wearable developer to ensure data accuracy [45].

Although regular calibration might be a burden, understanding its value would encourage patients to do it correctly [55]. However, considering the possibility for error to arise from the manual calibration of CGM devices with glucometers, an automatic calibration mechanism could be preferable.

Moreover, the wearable developers could conduct dynamic testing of the products. Clinicians want more collaboration with wearable developers to define strategies for continuous wearable assessment that can be achieved through various RPM interventions. Ideally, there should be a consensus among clinicians and wearable developers regarding guiding patients on the frequency of and providing instructions on calibration based on clinical principles.

#### Validation Results

Each of the 3 recommendations about PGHD accuracy was rated as “essential” (each one reached an aggregated 60% agreement as being important to very important).

### PGHD Completeness

Incomplete PGHD may be a result of technical or behavioral issues. Battery failure, wearable dysfunction, lack of synchronization in different time zones, internet disconnection, or patient’s neglect are among the accidental causes of insufficient PGHD. This may compel clinicians to reorder data collection. Moreover, there might be deliberate data omissions for both manual and automatic entries because of demotivation, lack of digital literacy, body pain, or perception of having no changes owing to seeing similar trends over time [61].

Lack of continuous follow-up may also result in incomplete data. Different health care settings define data sharing time frames differently in the remote monitoring of the same health condition; this can create confusion in patient and clinician communication. Lack of continuous interaction with patients during remote monitoring could result in a lack of engagement in self-care and motivation to collect data [62]. None of the published RPM studies have reported an approach to identify the exact reason for data incompleteness.

#### Recommendation 7: A Protocol Should Be Available to the Patient and Clinician That Defines PGHD Downtime, That Is, the Time Range During Which It Is Acceptable if the Wearable Is Not Collecting Data

Given the constant automated sensing capabilities of wearables, it is unclear whether patients are required to wear the devices continuously in different RPM programs to provide sufficient data for their care planning. There was lack of awareness among PGHD stakeholders in our studies on standardizing “downtime” when patients can stop data collection in different RPM programs. From clinicians’ point of view, a CGM wearable should be worn for at least 80% of the RPM period so that it can provide complete data for interpretation and decision-making. However, it is thought to be burdensome for patients to have to wear the device day and night and calibrate it frequently [55]. New generations of wearables seem to address “downtime” by letting patients turn off the device. Alerts could be designed in wearables to help clinicians discuss the reasons for incomplete data with patients.

As mentioned in the *PGHD Accuracy* section, clinicians would prefer focusing on data trends over time rather than single data points; therefore, some degree of missing data is acceptable [51]. Some clinicians do not see PGHD completeness as fundamental for sound decision-making [63]. However, it is important to know the extent of the impact of incompleteness on data interpretation and decisions made for patient care in the remote monitoring of different diseases [51,64]. Having a predefined and transparent downtime protocol on which the patient and clinician agree could clarify the completeness of PGHD [65].

#### Recommendation 8: A Protocol Should Be Available to the Patient and Clinician for Resuming PGHD Collection When the Acceptable Downtime Period Is Exceeded

Applying the acceptable time frame in which patients can stop collecting PGHD cannot be thoroughly understood unless patients are aware of when to resume data collection.

However, this might not happen if patients forget to do so. Moreover, owing to a lack of technical infrastructure for the real-time transmission of data from outside to inside the health care setting, clinicians are not aware of the missing data during data collection and thus are unable to alert patients to resume data capture [66,67]. This could be considered in the wearable design; for instance, wearables can provide patients the ability to set an alarm based on the predefined acceptable downtime schedule.

#### Recommendation 9: Annotation Function Should Be Available to the Patient in Order to Provide Context for Any Period of Missing PGHD

Any missing data need to be supplemented by contextual information to help clinicians identify the causes and discuss them with patients [63]. Contextual information about the missing data can help clinicians understand whether the problem was technical, behavioral, or related to the process of data transmission. Incomplete data in themselves do not explain the circumstances that led to their incompleteness [51].

Although some wearables were reported to provide notification of missing data, they still lack contextual information. This could place a burden on patients to be constantly attentive to record the causes of incompleteness. Innovative mechanisms could be designed to increase the interaction between the device and the wearer to record contexts for the incomplete data in a real-time or on a daily basis. The annotation feature that was mentioned in the *PGHD Accuracy* section is equally important to enable patients to enter information regarding missing data [56].

#### Validation Results

Each of the 3 recommendations about PGHD completeness was rated as “essential” (all reached an aggregated 60% agreement as being important to very important).

### PGHD Consistency

PGHD consistency is characterized by the ability to compare PGHD of one measurement from different devices as well as the ability to relate PGHD to the corresponding conventional clinical measurement.

Various wearables and associated mobile apps and web portals used in RPM programs may not represent data in a consistent manner. PGHD inconsistency can happen during data collection, transmission, and review, which immensely impacts data presentation that may have not been thoroughly recognized by PGHD stakeholders.

#### Recommendation 10: PGHD From Wearables Should Be Collected Based on Clinically Accepted and Structured Data Definitions and Standard Formats

Both consumer and medical wearable platforms may fail to represent data in clinically standardized formats [30,68,69]. Nonstandard presentation can result in confusion in data interpretation and inability to discern whether PGHD reports show normal or abnormal trends [51]. The standardization of health data elements is intended to define what data are to be collected, decide on how the collected data should be represented, and specify how the data should be encoded for transmission [70].

Collecting PGHD from different types and brands of wearables, each with its own data presentation format, could result in inconsistent reports [49,51]. Most of the recent PGHD-related policies advise developing standardized formats for PGHD collection that align with the clinical data standards, which are defined as protocols, terminologies, and specifications that are used during data management stages [23,50,54,71,72].

To ensure consistent definitions and formats for PGHD, 2 approaches should be considered: PGHD consistency at data collection and PGHD consistency at the data processing stage. Patients may need to be advised to collect PGHD from one type or brand of wearables for the remote monitoring of a particular health condition to provide consistent reports. Clinicians in our studies and others [62] preferred to give patients autonomy over device selection and stated that patients should have the right to select a convenient and easy-to-use wearable device. Nonetheless, because PGHD cannot be further filtered by information systems within a health care setting to fix the inconsistencies that emerge from collecting data in different formats, data that are presented for review might be difficult to interpret. Inconsistent reports at the data review stage are a consequence of collecting data from disparate wearable platforms. The second approach is to standardize PGHD at the data processing stage regardless of the wearable used in data collection. In this case, robust technical infrastructure needs to be in place to allow gathering PGHD from different wearables and their apps and portals in one database to filter and process data and present standardized reports that are similar to clinical data presentation formats.

Universally accepted data definitions and data exchange formats are required to facilitate effectual data transfer. Data should be codified according to the known clinical standards. In addition, ontologies that could aggregate and enrich PGHD with definitions, synonyms, and term relationships can be developed to provide standardized formats and make data semantically exchangeable [73].

#### Recommendation 11: PGHD From Wearables Should Be Integrated Into the Patient’s Clinical Care Record

Lack of PGHD integration with electronic medical record (EMR) systems is another barrier to PGHD consistency [30,39,74-76]. Current RPM programs are project oriented and not embraced in routine clinical practices. Moreover, most current EMR systems are not designed to seamlessly gather various types of data from outside the clinical setting in a straightforward manner [77]. PGHD should be combined with the patient’s clinical record to identify potential correlation with past conditions and be used in future interventions [30,46,54].

Despite the clinicians’ preference for the patients to choose their own brand of wearable, PGHD integration with EMRs constrains the selection of wearable. Patients should only use wearables in RPM that follow the interoperability standards used in the health care setting.

Most policies addressed the necessity of integrating PGHD with EMRs [23,26,35,54], but few provided specific suggestions. For example, the American Medical Association’s best practices for digital health implementation recommend that standard communication templates be designed before implementing RPM intervention to ensure consistency in data documentation during the whole process [72]. Therefore, PGHD integration with EMRs might be facilitated by modifying the EMRs, developing external dashboards, or limiting the choices of the brand of wearable used for data collection.

#### Recommendation 12: PGHD From Wearables Should Be Consistently Exchanged Inside and Between Clinical Settings

In addition to the need for standardized formats and integration with EMRs, PGHD exchange inside and outside the health care setting needs to be consistent by following health data exchange protocols.

If >1 health care setting is actively represented in an RPM program for one disease cohort or different departments implement RPM in one health care setting, data should be exchanged consistently to be understandable by different clinicians. This requires standardized formats for various types of PGHD.

Interoperability initiatives developed by the Australian Digital Health Agency [78] defined standards to facilitate data and information exchange and provided compliance mechanisms in connected health programs. These standards are broad and cover both PGHD and clinical data collected from outside and within health care settings. More specific interoperability standards were introduced by the Personal Connected Health Alliance with its Continua guidelines for data interoperability in personal connected health devices [79]. These initiatives need to be tested in various RPM programs to assess the consistency in data exchange.

#### Validation Results

recommendations 10 and 11 about PGHD consistency were rated as “essential” (they reached an aggregated 60% agreement as being important to very important), whereas recommendation 12 was rated by 9 (90%) of the 10 participants and reached less than 60% agreement for inclusion in the defined categories.

### PGHD Interpretability

Interpretability is affected by the way in which PGHD are presented as well as by the availability of contextual information regarding PGHD.

Not understanding the presented data can reduce patients’ and clinicians’ motivation for data collection and review [31,80,81]. Challenges of PGHD interpretation can occur at any stage of data management. This aspect was mentioned as the most challenging feature in our studies.

#### Recommendation 13: PGHD From Wearables Should Be Accompanied by Contextual Data That Are Clinically Important to Patient Management

The increasingly high volume and dynamically changing nature of PGHD make it difficult and time-consuming to gain a holistic view of a patient’s status from the data alone [31,74,82].

Most PGHD are not supplemented by contextual information about the circumstances in which the data were collected. Lack of context can lead to misunderstanding; misinterpretation; and, consequently, unsound decisions [80]. For example, when a clinician tries to discern a pattern in a processed PGHD report, it may be unclear whether a graph showing a lack of activity reflects the patient’s demotivation, a problem in the wearable’s function, or a medication interruption [56]. In this situation, relying on the patient’s verbal expression without recorded contextual information is not sufficient to draw an understanding of the patient’s situation per trend.

Similar to its application in PGHD completeness, context for PGHD is important for the data review stage so that clinicians can understand what the patient was doing at the point of data collection [31,52,74,83]. Among the wearables studied in this project, only CGM devices allowed the manual capture of limited contextual data—such as those about mood and exercise—in cases where the automatically captured data were reported to be erroneous or incomplete. PGHD from medical wearables can be contextualized by data that are automatically captured from consumer wearables [80,84]. However, no mechanism exists to integrate these 2 types of wearables in RPM, and there is uncertainty about which contextual data are more relevant to patient care. In addition, the ability to understand and interpret contextual information is still beyond clinicians’ expertise [85].

More collaboration between patients, clinicians, and wearable developers is needed to identify what contextual data need to be collected for each health condition and whether these data elements should be incorporated within the wearable design or require wearable integration.

#### Recommendation 14: Dynamic Visual Representation as Well as a Static Snapshot (Such as in PDF Format) of PGHD From Wearables Should Be Available to the Patient and Clinician

Clinicians review the processed PGHD reports either from the patient’s or clinician’s portal that the wearable developers design for them with static visualization of data that can be downloaded in a PDF format.

Having a snapshot of all the data collected over time provides a summary of the patient’s status, but as the amount of PGHD increases, such a report could be progressively more complex for their clinician to interpret [86]. Designing interactive visualization tools based on clinicians’ needs can result in easy PGHD interpretation [52,76,82,87].

Interactive visualizations could enable clinicians to highlight the most concerning areas and customize the reports based on different variables [87,88]. Interactive visualization supported by annotation capability can facilitate the cointerpretation of PGHD report, such as the ability to add highlights in a graph to detect changes. It is beneficial to patients to have a saved version of points and notes of what they and their clinicians identified and discussed for use in the next consultations. Likewise, in the subsequent clinic visit, the clinician could readily recollect what the previous consultation was focused on, which helps recognize patterns and set efficient care plans [56]. A dynamic and interactive visualization could also layer PGHD displays based on clinicians’ preferences. Studies have shown that different layers of data presentation, such as a holistic summary, an individual data summary, and detailed individual data, support the comprehensive interpretation of PGHD [52,76,82].

Notwithstanding, it is a challenge for wearable developers to design data presentation formats that please all clinicians with varying levels of digital health literacy. Collaboration among PGHD stakeholders is needed to determine who should design interactive dashboards for PGHD presentation, whether the dashboards should be implemented within EMRs or somewhere else in the health care setting, and how the reports should be presented to the patient and clinician to inform shared understanding and decision-making.

#### Recommendation 15: Alerts Should Be Sent to the Patient During PGHD Collection by the Wearable When Data Are Outside the Acceptable Range, Accompanied by Clinical Advice on Action to Take

In addition to clinicians’ interpretation of PGHD, it is important to ensure that patients can also interpret the data correctly. Not understanding PGHD can reduce the motivation to continue data collection. Efforts toward changing the patients’ roles from passive participants to active players in RPM require patients to understand PGHD and make necessary changes during data collection [89].

However, wearables do not provide understandable contextual information on PGHD. Most wearables display PGHD without further explanations of their meaning, the normal range, what will happen to the patient’s health status if their measurements are out of range, or what actions the patient could take if their measurements are out of range. This is problematic if the patient cannot immediately communicate with their clinician when they see significant changes occur in their data trends and do not know what action to take.

There are alarms embedded in some types of consumer wearables that notify out of range measurements [85]. Although PGHD from these tools may not require urgent actions, such features could improve patients’ interpretations of the data and better inform and influence behavior change.

Some medical wearables are equipped with a feature that alarms when the raw data go outside the normal range. Devices without this feature could be dangerous to a patient’s health, as immediate medical action may not be undertaken when needed. Nevertheless, the questions of to what extent patients could interpret PGHD augmented with contextual information during RPM without a clinician’s intervention and how sound a patient’s decision would be based on the interpretation are largely unexplored.

Cointerpretation of PGHD improves the shared understanding of data reports and generates an additional layer of meaning for PGHD in patient care plans [90]. These strategies particularly depend on patients’ and clinicians’ training and collaboration with the wearable developers to improve PGHD presentation design and interpretation.

#### Validation Results

Each of the 3 recommendations about PGHD interpretability was rated as “essential” (reached an aggregated 60% agreement as being important to very important).

### PGHD Relevancy

PGHD relevancy is characterized in various manners depending on the scope and coverage of data for each health condition. The values of conventional clinical data collected inside the health care setting are defined based on the standards of care. However, PGHD include a wide range of heterogeneous and new types of data whose relevancy to the monitored health condition might be unclear. Only 1 common theme was found in the previous studies of this project for PGHD relevancy, which resulted in the recommendation discussed next.

#### Recommendation 16: There Should Be a Shared Understanding Between the Patient and Clinician of Relevant Data for the Disease Based on the Standards of Care, and Make Sure That All the Relevant Data Are Collected

PGHD relevancy was perceived as the most distinguishing DQM factor in using PGHD from consumer wearables versus those from medical wearables in RPM, and its lack was perceived as the most predominant barrier to the adoption of PGHD in clinical practice [91]. Patients and clinicians might have different perspectives on which types of PGHD are relevant to patient care [31]. Patients’ enthusiasm to use a wide range of consumer wearables and collect new types of PGHD that have not been collected easily before (eg, heart rate, sleep quality, and activity level) increases their expectation from clinicians to review the data. By contrast, clinicians might not be convinced of the extent to which the data are relevant to the health condition and supplement the clinical data collected from medical wearables to provide a better picture of patients’ status.

Clinicians involved in this project along with other studies indicated that PGHD from consumer wearables have not yet been proven to correlate with most health conditions and that they are different from other clinical data in terms of clinical value [39,76,82].

Even if PGHD are collected from medical wearables, it would still be challenging to identify whether all the data are relevant to the specific health condition. Conversely, some wearables cannot capture all the relevant PGHD; therefore, important relevant data might be missed, which might lead to incorrect decisions about patient care [76,83]. The need for the collection and analysis of relevant data was addressed by recent PGHD-related policies [23,54,72]. Only 2 clinical guidelines developed to address the details and level of relevancy of PGHD collected from wearable devices for the remote monitoring of patients with diabetes and those with cardiac arrhythmia were identified [60,65]. More guidelines are needed to determine relevant PGHD for the remote monitoring of each health condition.

#### Validation Results

Half of the participants rated PGHD relevancy recommendation as very important (40%) to important (10%), whereas 40% addressed it as moderately (30%) to slightly important (10%).

### PGHD Timeliness

PGHD timeliness is characterized by the timing and frequency of PGHD availability to patients and clinicians.

#### Recommendation 17: PGHD From Wearables Should Be Available to the Patient Within a Timeframe (Continuously to Periodically) According to the Standards of Care of Diseases

Our findings showed that the timing of PGHD availability to patients was overlooked. Although accessing data during data collection is critical when a decision needs to be made, some wearables do not provide real-time PGHD access to patients during data collection. As discussed in the *PGHD Accessibility* section, depending on the health condition and the clinical purpose of RPM, PGHD presentation to patients in real time might be deliberately disabled by clinicians. However, studies have shown that accessing real-time data from the wearable increased patients’ awareness of the wearable’s function, further engaged them in self-care, and enhanced shared decision-making [92-94].

As PGHD collection in RPM are led by clinicians, patients may not be fully aware of their rights in accessing PGHD at data collection and how it might impact their safety. PGHD access in real-time or periodic mode needs to be defined according to the standards of care of the health condition [65]. RPM interventions could be designed based on patient-centered care models where time frames could be established so that patients can access their data during data collection to make a proper decision, change their behavior, or immediately contact the clinician.

#### Recommendation 18: PGHD From Wearables Should Be Available to the Clinician Within a Timeframe (Continuously to Periodically) According to the Standards of Care of Diseases

The most challenging issue reported about PGHD timeliness is the lack of clinicians’ access to data in real time [39,77,83]. As PGHD are not yet integrated with EMRs, it is difficult and time-consuming to frequently receive the data and follow-up with patients. PGHD integration with EMRs would provide possibilities for generating alerts on newly added PGHD in the EMR system in a real-time or near real–time basis so that clinicians can be updated on a patient’s status and provide prompt feedback [83]. Notwithstanding, the technical integration by itself is not the ultimate solution. PGHD need to be fully incorporated into clinical workflows such that clinicians could receive data based on predefined protocols and be able to provide timely advice to patients [95]. Timely access to PGHD without immediate feedback to patients would lead to patient demotivation on data sharing [96]. However, RPM interventions may have different protocols for PGHD availability to clinicians. In some cases of remote monitoring of patients with diabetes, clinicians remotely obtain PGHD reports from patients during data collection, whereas in others, they see the report after data collection during the clinic consultation. Having predefined protocols might facilitate clinicians’ access to PGHD within a specific time frame.

#### Recommendation 19: A Timeframe for Sharing PGHD From Wearables Should Be Available to the Patient and Clinician

As noted earlier, RPM programs apply disparate time frames for PGHD sharing. This way of accessing data can be challenging. Patients who access data in real time may also need to receive a clinical advice immediately, whereas data are not available to clinician in the same time frame. Frequent data sharing during data collection could help recognize some behaviors that might not be identified when the collection period is finished.

PGHD need to be available when there is an urgent need for clinical advice so that the patient can change the way of data collection or their behavior accordingly [30,97]. However, findings showed that this depends on the health condition; for example, the guideline on using wearables in cardiac RPM emphasized that these services should not be mistaken with acute care; therefore, there is no urgent need for real-time feedback [65]. Hence, based on the health context, having transparent protocols on data sharing could help clinicians review PGHD and set patients’ expectations for data transmission and feedback [76].

As different RPM programs may need different approaches on data timeliness based on the standards of care, there should be a single time frame defined for the remote monitoring of each health condition to ensure consistency among the programs.

#### Validation Results

All of the recommendations about PGHD timeliness were rated as “essential” to the safety of and quality of care in RPM (reached an aggregated 60% agreement as being important to very important).

### A Staged Model of Quality Management of PGHD in RPM

**Figure 2:**
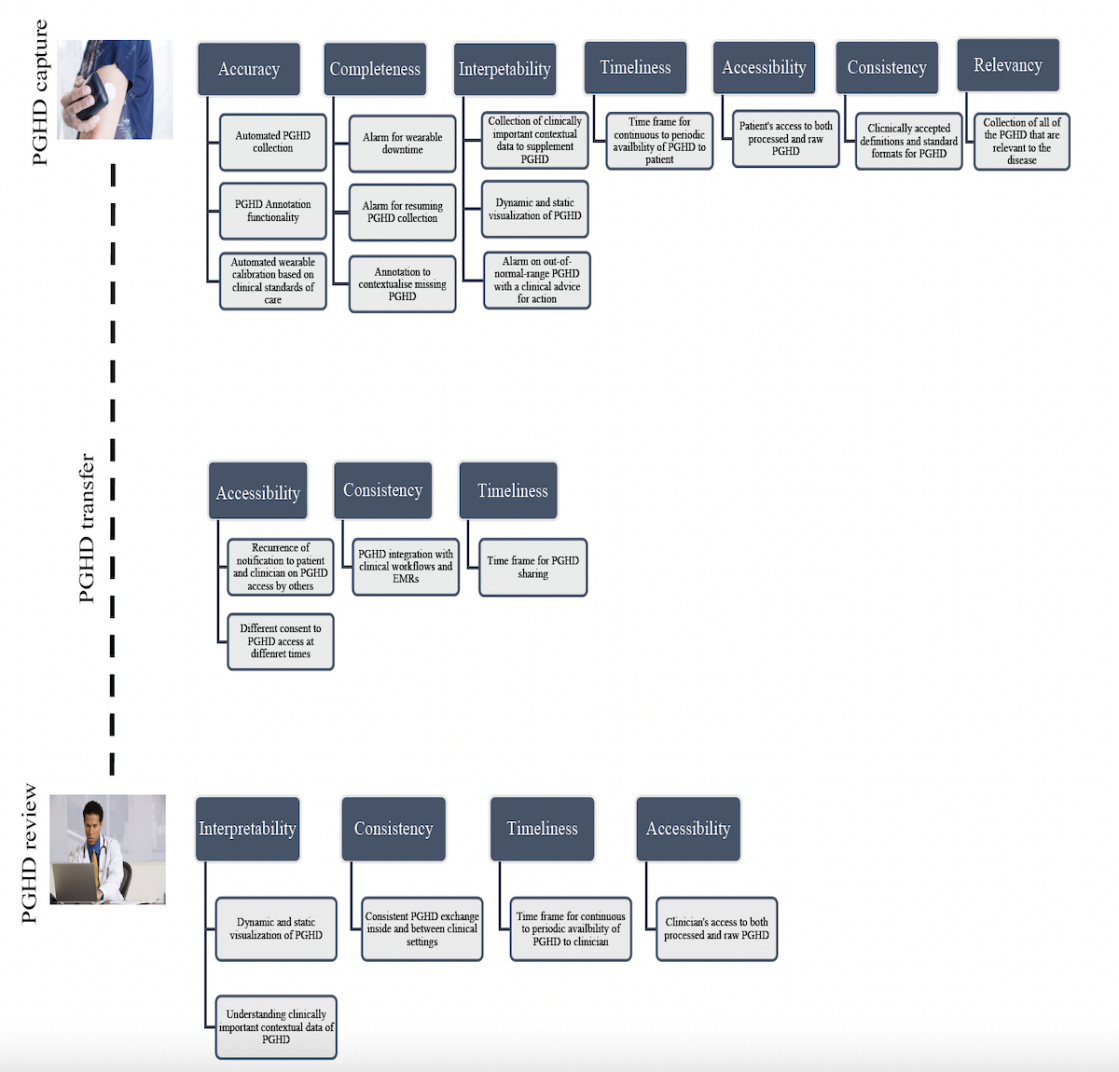
Recommendations for the quality management of patient-generated health data (PGHD) at the 3 stages of PGHD management. EMR: electronic medical record.

Figure 2 illustrates the recommendations according to the importance of their consideration at different stages of data management. This model can assist PGHD stakeholders in understanding what DQM actions need to be taken to efficiently collect, manage, and use PGHD in RPM.

As shown in Figure 2, all DQM aspects of PGHD require attention at the data collection stage. It indicates that the quality management of PGHD is critical when data are collected outside the clinical environment under patients’ or their caregivers’ supervision. Data access, consistency, and timeliness were the most critical DQM aspects to be considered during PGHD transmission from the patient to clinician. These 3 aspects along with PGHD interpretability require emphasis when clinicians review the data reports for shared decision-making and creating patient care plans.

As there are interconnections among DQM aspects, this model indicates that collaborative actions need to be undertaken by different PGHD stakeholders to practice DQM and ensure high-quality PGHD in RPM.

## Discussion

### Principal Findings

This paper presented the development of 19 recommendations for 7 DQM aspects of PGHD collected from wearable devices in RPM programs. The guideline aims to assure that high-quality data are collected, managed, and used in RPM programs to improve the safety and quality of these programs and enhance PGHD fitness for use in routine clinical practice.

The guideline was constructed by following 4 steps of guideline development process through 5 qualitative studies. The guideline was then conceptualized to address 3 main concepts: PGHD management process, DQM aspects of PGHD, and sociotechnical issues that influence the quality management of PGHD during the data management process.

The DQM guideline for PGHD is distinguished from conventional DQM guidelines for clinical data in several ways: (1) it emphasizes the need for action corresponding to each DQM aspect at each stage of PGHD management; (2) it considers both external sociotechnical factors and internal organizational factors that impact the quality management of PGHD in RPM; (3) it recognizes patients’ and clinicians’ needs for each DQM aspect of PGHD, as the key PGHD stakeholders in RPM. This guideline is intended mainly for using PGHD for patient care. It is anticipated that the guidelines can also be used alongside conventional DQM guidelines for clinical data to assure PGHD quality and when these data are integrated into EMR systems.

To effectively apply the guideline in the remote monitoring of various health conditions, wearable devices should not be considered as stand-alone tools that work in isolation. Instead, they should be looked at as one component of a bigger ecosystem where different stakeholders interact with each other, with the devices, data, technical infrastructure of the health care setting, and standards to ensure that high-quality PGHD are collected, managed, and used for patient care [3]. The guideline can be best applied when RPM is implemented for >1 health condition across the health care system and when PGHD are collected from >1 type of wearable device and system interconnections are facilitated [98]. Also, realizing the value of high-quality PGHD for patient care can potentially blur the reliability distinctions between the 2 types of wearables, consumer and medical wearables. Being approved by regulatory agencies as a medical grade wearable does not ensure that the PGHD from it achieve a satisfactory level of quality. PGHD from consumer wearables are rarely used in current RPM services, and the research findings mainly included PGHD from medical wearables, so unseen challenges might exist to the quality management of PGHD from consumer devices. Advances in the capabilities of consumer devices and patients’ and clinicians’ accessibility to them are likely to see greater crossover between medical and consumer wearables in the future.

The DQM guideline for PGHD in RPM cannot be successfully implemented and used if the health system does not address the factors listed in Textbox 2.

The implementation of PGHD quality management in RPM can benefit the health care system and those who are considered the stakeholders of PGHD and who might advantage from the incorporation of these data into clinical practice, including the groups listed in Textbox 3.

**Considerations for the implementation of the data quality management (DQM) guideline.**

- Policies: clinical, technical, and organizational policies need to be in place in parallel with the guideline for the quality management of patient-generated health data (PGHD) to increase the likelihood that PGHD will be trustworthy for use in clinical care.
- Technical infrastructure: PGHD from wearables used in remote patient monitoring (RPM) programs are not yet integrated routinely into electronic medical record systems or able to flow securely across the health care system; both factors are key barriers to using PGHD in clinical care. The guideline can be best applied when a technical infrastructure is established to follow the recommendations for the systematic management, interactive and standardized presentation, and consistent exchange of PGHD and standardized and timely access of PGHD reports to patients and clinicians.
- Digital health literacy: understanding the quality management of PGHD requires sufficient digital health literacy among all PGHD stakeholders. The conceptual model shows that all DQM aspects need action in the stage when patients collect PGHD, emphasizing patients’ need for literacy to understand DQM. Training delivered to patients and caregivers could enhance their engagement in the collection and management of high-quality data. Moreover, RPM teams could be expanded to include professionals who could provide DQM advice and support to clinical stakeholders.
- Collaboration: without collaboration among all PGHD stakeholder groups, the guideline recommendations cannot be implemented effectively in RPM. In addition to the stakeholders that were involved in this project, other stakeholders such as payers, policy makers, and health care administrative need to collaborate with the RPM team to understand the implementation requirements of the DQM guideline of PGHD. Continuous collaborative efforts to evaluate wearable devices, PGHD, and the data management processes could provide the health system with high-quality data that are fit for clinical care, population health management, and secondary uses.

**Stakeholders who can benefit from patient-generated health data (PGHD) quality management in remote patient monitoring (RPM)**

- Patients and carers: patients will be able to collect high-quality data to manage their conditions. They will learn to correctly use digital health devices and collect high-quality PGHD that could be clinically valuable and used optimally in clinical care. Moreover, by collecting relevant and quality-assured PGHD and sharing them with a single RPM system, patients’ role in RPM could be changed from passive to active participants, strengthening their interactions with clinicians, improving shared decision-making, and better engaging them in their health self-management.
- Clinicians: the pandemic has increased clinicians’ awareness of the potential uses of RPM and PGHD. However, they need a reliable and convenient way to determine the utility of PGHD from patients, based on how and when these data are collected and reported and how and when they and others can access and interact with the data. The use of PGHD quality management recommendations enable clinicians to assess the quality of available data to support a patient consultation and how these data can form a valuable basis for efficient shared decision-making. Through this, they could optimize their focus on PGHD during and between patient visits.
- Health information professionals: health information professionals are nonclinical staff, including health informaticians, health information managers, and other experts who monitor data and information management within the health care system. The implementation of the guideline could bring new responsibilities and roles for these professionals. For example, these experts can play a critical role in defining new approaches to manage PGHD and use their skills to work collaboratively on data integration and management. In addition, they could act as gatekeepers before PGHD become available to clinicians and filter and analyze the data to provide the most meaningful information for clinicians.
- Health care organizations: for maintaining RPM after the pandemic, health care organizations need to be sure that PGHD from different digital health tools will support safer, higher-quality, and faster decision-making, based on more persuasive patient-clinician communication, leading to more effective and efficient health outcomes. The implementation of the RPM system driven by the PGHD quality management guideline could assist health care organizations in taking a standard approach to data integration, quality assurance, and risk management of these data to increase their trustworthiness for use in patient care. It may also provide health care organizations with strategies to think about the required infrastructure, policies, human resources, and potential collaborations with other parties to enhance the use of PGHD in clinical practice.
- Digital health technology companies: medical device companies may be alerted to the existing unrecognized problems in ensuring the quality of data from their proprietary devices and offer solutions to overcome these. This may address the need for better synergies with the existing health data standards and health information system architectures to enable data sharing from various devices. Consumer device companies may also realize the gaps in ensuring the quality of data from their devices. This could assist in developing higher-quality devices with user-friendly and validated data handling solutions that are capable of being integrated more readily into the existing clinical information systems.
- Other beneficiaries: findings from this research may also have indirect benefits, including providing insights into PGHD features and functions to the developers of electronic clinical information systems, such as patient records and point-of-care decision-support. These insights may inform the development of health policies and regulation of PGHD, including their use in research and public health, and could also provide more research opportunities in this area considering other kinds of PGHD and solutions for the further use of such data in broader contexts.

### Limitations

PGHD collection for self-management purposes without clinical use was out of the scope of this research. Moreover, this study did not concentrate on the concept of data quality as used in the biomedical engineering domain, such as the accuracy of the formula or algorithms embedded in wearables. We also limited the exploration of PGHD to their use in direct patient care, engaging with the stakeholders in this kind of use, and excluded the secondary uses of data, such as in outcomes research, surveillance, reimbursement strategies, and purposes other than patient care.

The recommendations of the DQM guideline of PGHD were defined at a high level. They would benefit from the addition of details that specify the roles and responsibilities of different stakeholder groups. This would require the guideline to be investigated more deeply with participation from different stakeholder groups to identify further considerations in different contexts.

The guideline might be questioned as not being specific to one health condition when it is known that RPM initiatives are distinctive in different contexts of care. However, it was extracted from the RPM initiatives for 3 chronic conditions that showed similarities and commonalities in the quality management of PGHD. It is worth noting that digital health implementation is moving toward focusing on the patient as a whole rather than the disease. Therefore, RPM initiatives, as well as the data they collect, could also shift their focus from a specific disease and wearable to services for the integrated management of all the health conditions that a patient might have [72]. Nevertheless, for further exploration, the guideline can be implemented in each disease-based RPM to provide more specific recommendations based on particular needs. Understanding what makes PGHD more reliable for shared decision-making can motivate PGHD stakeholders to have a shared understanding of the value of these data and use them more efficiently to achieve better health outcomes.

### Comparison With Prior Work

Research on the adoption, integration, and evaluation of RPM, wearables, and PGHD in clinical practice is rapidly growing [99-106], particularly during the COVID-19 pandemic, when many RPM initiatives were implemented around the world.

However, a few studies focused on PGHD quality [51,62,69,107] had aims and scopes that were different from those of our research. This is the first study of its kind that adapted 7 common aspects of DQM and investigated them in PGHD context during PGHD management stages. It also involved various groups of international PGHD stakeholders to share their experiences, concerns, and expectations regarding the quality management of PGHD and constructed and validated a set of recommendations as a novel guideline. This process helped reach a consensus among the participants on the recommendations they could follow to effectively collaborate for better patient care.

### Conclusions

Although the quality of PGHD is addressed as a vital factor in increasing their reliability in clinical decision-making, this research is the first of its kind to explore the quality management of PGHD through 7 aspects during data management stages. The guideline developed in this research provides a major step forward in this regard. It gives PGHD stakeholders a framework for improving the quality management of PGHD collected and used in RPM underpinned by collaboration.

## Appendix

Multimedia Appendix 1 The synthesis of the findings of this project’s previous studies and the resulting themes that constructed the recommendations.

## Abbreviations

CGM: continuous glucose monitoring
DQM: data quality management
EMR: electronic medical record
PGHD: patient-generated health data
RPM: remote patient monitoring

## References

[1] Field MJ, Grigsby J (2002). Telemedicine and remote patient monitoring. JAMA.

[2] Taiwo O, Ezugwu AE (2020). Smart healthcare support for remote patient monitoring during covid-19 quarantine. Inform Med Unlocked.

[3] Azodo I, Williams R, Sheikh A, Cresswell K (2020). Opportunities and challenges surrounding the use of data from wearable sensor devices in health care: qualitative interview study. J Med Internet Res.

[4] Kraef C, van der Meirschen M, Free C (2020). Digital telemedicine interventions for patients with multimorbidity: a systematic review and meta-analysis. BMJ Open.

[5] Abdolkhani R, Gray K, Borda A, DeSouza R (2019). Patient-generated health data management and quality challenges in remote patient monitoring. JAMIA Open.

[6] Tayi GK, Ballou DP (1998). Examining data quality. Commun ACM.

[7] WHO Regional Office for the Western Pacific, World Health Organization. Regional Office for the Western Pacific (2003). Improving Data Quality A Guide for Developing Countries.

[8] Data duality frameworknterprise business intelligence solutions; 2013. The Australian Capital Territory Health.

[9] (2015). Data Quality Management Model (2015 Update) - Retired. The American Health Information Management Association.

[10] Andrews TN (2017). Guide to the health facility data quality report card. SILO.

[11] (2016). Data quality assessment and improvement strategies. The European Institute for Innovation through Health Data.

[12] Canadian Institute for Health Information (2009). The CIHI Data Quality Framework.

[13] Woolf S (1992). Practice guidelines, a new reality in medicine. Arch Intern Med.

[14] (2012). The guidelines manual Process and methods. National Institute for Health and Care Excellence.

[15] Hanson D, Hoss B, Wesorick B (2008). Evaluating the evidence: guidelines. AORN J.

[16] AGREE Collaboration (2003). Development and validation of an international appraisal instrument for assessing the quality of clinical practice guidelines: the AGREE project. Qual Saf Health Care.

[17] Field M, Lohr K, Committee on Clinical Practice Guidelines, Institute of Medicine (1992). Guidelines for Clinical Practice From Development to Use.

[18] Weiskopf NG, Bakken S, Hripcsak G, Weng C (2017). A data quality assessment guideline for electronic health record data reuse. EGEMS (Wash DC).

[19] Abdolkhani R, Borda A, Gray K (2018). Quality management of patient generated health data in remote patient monitoring using medical wearables - a systematic review. Stud Health Technol Inform.

[20] Abdolkhani R, Gray K, Borda A, DeSouza R (2020). Quality assurance of health wearables data: participatory workshop on barriers, solutions, and expectations. JMIR Mhealth Uhealth.

[21] Major C, Savin-Baden M (2011). Integration of qualitative evidence: towards construction of academic knowledge in social science and professional fields. Qual Res.

[22] Hsu C, Sandford B (2007). The delphi technique: making sense of consensus. Pract Asses Res Eval.

[23] (2018). Our data-driven future in healthcare. Academy of Medical Sciences.

[24] Turakhia M, Desai M, Hedlin H, Rajmane A, Talati N, Ferris T, Desai S, Nag D, Patel M, Kowey P, Rumsfeld JS, Russo AM, Hills MT, Granger CB, Mahaffey KW, Perez MV (2019). Rationale and design of a large-scale, app-based study to identify cardiac arrhythmias using a smartwatch: The Apple Heart Study. Am Heart J.

[25] (2017). Manufacturers sharing patient-specific information from medical devices with patients upon request 2017. U.S. Food & Drug Administration.

[26] (2017). Redefining our picture of health: towards a person-centered integrated care, research, wellness, and community ecosystem. American Medical Informatics Association.

[27] Art. 13 GDPR Information to be provided where personal data are collected from the data subject. Intersoft Consulting.

[28] (2016). Best practices for consumer wearables and wellness apps and devices 2016. Robert Wood Johnson Foundation.

[29] Chung C, Dew K, Cole A, Zia J, Fogarty J, Kientz J (2016). Boundary negotiating artifacts in personal informatics: patient-provider collaboration with patient-generated data. Proceedings of the 19th ACM Conference on Computer-Supported Cooperative Work & Social Computing.

[30] Rickardsson I (2016). Patient-generated health data: professionals' opinions and standardized data transfer. Digitala Vetenskapliga Arkivet.

[31] West P, Giordano R, Kleek M, Shadbolt N (2016). The quantified patient in the doctor's office:challenges and opportunities. Proceedings of the 2016 CHI Conference on Human Factors in Computing Systems.

[32] Bellekens X, Nieradzinska K, Bellekens A, Seeam P, Hamilton A, Seeam A (2016). A study on situational awareness security and privacy of wearable health monitoring devices. Int J Cyber Situational Awareness.

[33] Paul G, Irvine J (2014). Privacy implications of wearable health devices. Proceedings of the 7th International Conference on Security of Information and Networks.

[34] Castell S, Roinson L, Ashford H (2018). Future data-driven technologies and the implications for use of patient data. Ipsos MORI.

[35] (2019). Using remote patient monitoring technologies for better cardiovascular disease outcomes guidance. American Heart Association.

[36] (2018). 2018 Model privacy notice. The U.S. Office of National Coordinator for Health IT.

[37] (2018). Medical device cyber security Draft guidance and information for consultation. Australian Government Department of Health.

[38] Addonizio G (2016). The privacy risks surrounding consumer health and fitness apps, associated wearable devices, and HIPAA’s limitations. Law School Student Scholarship.

[39] Zhu H, Colgan J, Reddy M, Choe E (2016). Sharing patient-generated data in clinical practices: an interview study. AMIA Annu Symp Proc.

[40] Hernández N, Castro L, Favela J, Michán L, Arnrich B (2017). Data quality in mobile sensing datasets for pervasive healthcare. Handbook of Large-Scale Distributed Computing in Smart Healthcare. Scalable Computing and Communications.

[41] Ostherr K, Borodina S, Bracken RC, Lotterman C, Storer E, Williams B (2017). Trust and privacy in the context of user-generated health data. Big Data Soc.

[42] (2016). U.S. Department of Health and Human Services.

[43] (2018). New technologies that use patient data. The Academy of Medical Sciences.

[44] Sharko M, Wilcox L, Hong M, Ancker J (2018). Variability in adolescent portal privacy features: how the unique privacy needs of the adolescent patient create a complex decision-making process. J Am Med Inform Assoc.

[45] Engler R, Routh T, Lucisano J (2018). Adoption barriers for continuous glucose monitoring and their potential reduction with a fully implanted system: results from patient preference surveys. Clin Diabetes.

[46] Kakkanatt C, Benigno M, Jackson VM, Huang PL, Ng K (2018). Curating and integrating user-generated health data from multiple sources to support healthcare analytics. IBM J Res Dev.

[47] Badawy R, Raykov Y, Evers L, Bloem B, Faber M, Zhan A, Claes K, Little M (2018). Automated quality control for sensor based symptom measurement performed outside the lab. Sensors (Basel).

[48] Genes N, Violante S, Cetrangol C, Rogers L, Schadt E, Chan Y-F (2018). From smartphone to EHR: a case report on integrating patient-generated health data. NPJ Digit Med.

[49] Lewinski A, Drake C, Shaw R, Jackson G, Bosworth H, Oakes M, Gonzales S, Jelesoff NE, Crowley MJ (2019). Bridging the integration gap between patient-generated blood glucose data and electronic health records. J Am Med Inform Assoc.

[50] Robeznieks A (2019). Xcertia releases updated mobile health-app guidelines for comment. American Medical Association.

[51] West P, Van Kleek M, Giordano R, Weal M, Shadbolt N (2018). Common barriers to the use of patient-generated data across clinical settings. Proceedings of the 2018 CHI Conference on Human Factors in Computing Systems.

[52] Ryokai K, Michahelles F, Kritzler M, Syed S (2015). Communicating and interpreting wearable sensor data with health coaches. Proceedings of the 9th International Conference on Pervasive Computing Technologies for Healthcare.

[53] King RC, Villeneuve E, White RJ, Sherratt RS, Holderbaum W, Harwin WS (2017). Application of data fusion techniques and technologies for wearable health monitoring. Med Eng Phys.

[54] (2018). Conceptualizing a data infrastructure for the capture, use, and sharing of patient-generated health data in care delivery and research through 2024. Accenture Federal Services for the Office of the National Coordinator for Health Information Technology.

[55] Lawton J, Blackburn M, Allen J, Campbell F, Elleri D, Leelarathna L, Rankin D, Tauschmann M, Thabit H, Hovorka R (2018). Patients' and caregivers' experiences of using continuous glucose monitoring to support diabetes self-management: qualitative study. BMC Endocr Disord.

[56] Mentis H, Komlodi A, Schrader K, Phipps M, Gruber-Baldini A, Yarbrough K, Shulman L (2017). Crafting a view of self-tracking data in the clinical visit. Proceedings of the 2017 CHI Conference on Human Factors in Computing Systems.

[57] Davidson E, Simpson C, Demiris G, Sheikh A, McKinstry B (2013). Integrating telehealth care-generated data with the family practice electronic medical record: qualitative exploration of the views of primary care staff. Interact J Med Res.

[58] Shin M (2012). Secure remote health monitoring with unreliable mobile devices. J Biomed Biotechnol.

[59] Cappon G, Acciaroli G, Vettoretti M, Facchinetti A, Sparacino G (2017). Wearable continuous glucose monitoring sensors: a revolution in diabetes treatment. Electronics.

[60] Danne T, Nimri R, Battelino T, Bergenstal R, Close K, DeVries J, Garg S, Heinemann L, Hirsch I, Amiel SA, Beck R, Bosi E, Buckingham B, Cobelli C, Dassau E, Doyle FJ, Heller S, Hovorka R, Jia W, Jones T, Kordonouri O, Kovatchev B, Kowalski A, Laffel L, Maahs D, Murphy HR, Nørgaard K, Parkin CG, Renard E, Saboo B, Scharf M, Tamborlane WV, Weinzimer SA, Phillip M (2017). International consensus on use of continuous glucose monitoring. Diabetes Care.

[61] Shin G, Feng Y, Jarrahi M, Gafinowitz N (2019). Beyond novelty effect: a mixed-methods exploration into the motivation for long-term activity tracker use. JAMIA Open.

[62] Codella J, Partovian C, Chang H, Chen C (2018). Data quality challenges for person-generated health and wellness data. IBM J Res Dev.

[63] Rutjes H, Kersten-van DE, Willemsen M, IJsselsteijn W (2018). Tell-tale data: the value of self-tracked data for healthcare professionals. Proceedings of the 2018 Short Workshop on Next Steps Towards Long Term Self Tracking".

[64] Cai L, Zhu Y (2015). The challenges of data quality and data quality assessment in the big data era. Data Sci J.

[65] Slotwiner D, Varma N, Akar JG, Annas G, Beardsall M, Fogel RI, Galizio NO, Glotzer TV, Leahy RA, Love CJ, McLean RC, Mittal S, Morichelli L, Patton KK, Raitt MH, Ricci RP, Rickard J, Schoenfeld MH, Serwer GA, Shea J, Varosy P, Verma A, Yu C (2015). HRS Expert Consensus Statement on remote interrogation and monitoring for cardiovascular implantable electronic devices. Heart Rhythm.

[66] Wang E, Zhou L, Parmanto B, Watzlaf V, Abdelhak M (2018). Clinician's perceptions and expectations on a mHealth platform for supporting patient data integration and clinical service delivery: a case study in evidence-based communication rehabilitation. Proceedings of the Hawaii International Conference on System Sciences 2018.

[67] Jung S, Kim J, Hwang H, Lee K, Baek R-M, Lee H-Y, Yoo S, Song W, Han JS (2019). Development of comprehensive personal health records integrating patient-generated health data directly from Samsung s-health and apple health apps: retrospective cross-sectional observational study. JMIR Mhealth Uhealth.

[68] Botha M, Botha A, Herselman M (2014). Data quality challenges: a content analysis in the e-health domain. Proceedings of the 2014 4th World Congress on Information and Communication Technologies (WICT 2014).

[69] West P, Van Kleek M, Giordano R, Weal M, Shadbolt N (2017). Information quality challenges of patient-generated data in clinical practice. Front Public Health.

[70] (2004). Patient Safety: Achieving a New Standard for Care.

[71] Health information technology standards 2013. Public Health Data Standards Consortium.

[72] (2018). Remote Patient Monitoring Playbook. American Medical Association.

[73] Kim H, Lee S, Baik S, Kim J (2015). MELLO: medical lifelog ontology for data terms from self-tracking and lifelog devices. Int J Med Inform.

[74] Chung C, Cook J, Bales E, Zia J, Munson S (2015). More than telemonitoring: health provider use and nonuse of life-log data in irritable bowel syndrome and weight management. J Med Internet Res.

[75] Giordanengo A, Arsand E, Grottland A, Bradway M, Hartvigsen G (2019). Acceptance barriers of using patients’ self-collected health data during medical consultation. Proceedings of the 17th Scandinavian Conference on Health Informatics.

[76] Cohen D, Keller S, Hayes G, Dorr D, Ash J, Sittig D (2016). Integrating patient-generated health data into clinical care settings or clinical decision-making: lessons learned from project healthdesign. JMIR Hum Factors.

[77] Kumar R, Goren N, Stark D, Wall D, Longhurst C (2016). Automated integration of continuous glucose monitor data in the electronic health record using consumer technology. J Am Med Inform Assoc.

[78] (2018). Interoperability and connected healthcare in Australia early engagement paper. Australian Government Australian Digital Health Agency.

[79] (2017). Personal Connected Health Alliance.Interoperability design guidelines for personal connected health systems. Continua Design Guidelines®.

[80] Islind AS, Lindroth T, Lundin J, Steineck G (2018). From narratives to numbers: data work and patient-generated health data in consultations. Stud Health Technol Inform.

[81] Backonja U, Haynes S, Kim K (2018). Data visualizations to support health practitioners' provision of personalized care for patients with cancer and multiple chronic conditions: user-centered design study. JMIR Hum Factors.

[82] Kim Y, Heo E, Lee H, Ji S, Choi J, Kim J, Lee J, Yoo S (2017). Prescribing 10,000 steps like aspirin: designing a novel interface for data-driven medical consultations. Proceedings of the 2017 CHI Conference on Human Factors in Computing Systems.

[83] Mishuris RG, Yoder J, Wilson D, Mann D (2016). Integrating data from an online diabetes prevention program into an electronic health record and clinical workflow, a design phase usability study. BMC Med Inform Decis Mak.

[84] Holt J, Cusatis R, Asan O, Williams J, Nukuna S, Flynn K, Moore J, Crotty BH (2020). Incorporating patient-generated contextual data into care: clinician perspectives using the consolidated framework for implementation science. Healthc (Amst).

[85] Raj S, Lee JM, Garrity A, Newman MW (2019). Clinical Data in Context. Proc ACM Interact Mob Wearable Ubiquitous Technol.

[86] Austin R (2018). Picturing patterns in whole-person health: leveraging visualization techniques with structured consumer-generated mHealth data. Libraries Digital Conservancy.

[87] Feller D, Burgermaster M, Levine M, Smaldone A, Davidson P, Albers D, Mamykina L (2018). A visual analytics approach for pattern-recognition in patient-generated data. J Am Med Inform Assoc.

[88] Visualising health. Visualising Health Homepage.

[89] Petersen C (2018). Patient informaticians: turning patient voice into patient action. JAMIA Open.

[90] Van KJ, Bogers S, Rutjes H, Deckers E, Frens J, Hummels C (2018). Exploring the value of parent tracked baby data in interactions with healthcare professionals: a data-enabled design exploration. Proceedings of the 2018 CHI Conference on Human Factors in Computing Systems.

[91] Harle C, Listhaus A, Covarrubias C, Schmidt S, Mackey S, Carek P, Fillingim RB, Hurley RW (2016). Overcoming barriers to implementing patient-reported outcomes in an electronic health record: a case report. J Am Med Inform Assoc.

[92] Daley C, Chen E, Roebuck A, Ghahari R, Sami A, Skaggs C, Carpenter M, Mirro M, Toscos T (2017). Providing patients with implantable cardiac device data through a personal health record: a qualitative study. Appl Clin Inform.

[93] Mirro M, Daley C, Wagner S, Rohani Ghahari R, Drouin M, Toscos T (2018). Delivering remote monitoring data to patients with implantable cardioverter-defibrillators: does medium matter?. Pacing Clin Electrophysiol.

[94] Daley C, Toscos T, Mirro M (2019). Data integration and interoperability for patient-centered remote monitoring of cardiovascular implantable electronic devices. Bioengineering (Basel).

[95] Fajingbesi FE, Olanrewaju RF, Rasool Pampori B, Khan S, Yacoob M (2017). Real time telemedical health care systems with wearable sensors. Asian J Pharmaceutical Res Health Care.

[96] Healthcare coaching: multiplying the value of wearables and patient-generated health data 2018. fitbit Health Solutions.

[97] Ding H, Fatehi F, Russell A, Karunanithi M, Menon A, Bird D, Gray LC (2018). User experience of an innovative mobile health program to assist in insulin dose adjustment: outcomes of a proof-of-concept trial. Telemed J E Health.

[98] Williams R, Will C, Weiner K, Henwood F (2020). Navigating standards, encouraging interconnections: infrastructuring digital health platforms. Inform Commun Society.

[99] Smuck M, Odonkor C, Wilt J, Schmidt N, Swiernik M (2021). The emerging clinical role of wearables: factors for successful implementation in healthcare. NPJ Digit Med.

[100] Ye J (2021). The impact of electronic health record-integrated patient-generated health data on clinician burnout. J Am Med Inform Assoc.

[101] Turner K, Jo A, Wei G, Tabriz A, Clary A, Jim H (2021). Sharing patient-generated data with healthcare providers: findings from a 2019 national survey. J Am Med Inform Assoc.

[102] Petersen C (2021). Use of patient-generated health data for shared decision-making in the clinical environment: ready for prime time. Mhealth.

[103] Rosner B, Kvedar J, Adler-Milstein J (2021). Patient-generated health data earn a seat at the table: clinical adoption during the COVID-19 transition to telemedicine. JAMIA Open.

[104] Winter JS, Davidson E (2022). Harmonizing regulatory regimes for the governance of patient-generated health data. Telecommun Policy.

[105] Melstrom L, Rodin A, Rossi L, Fu JP, Fong Y, Sun V (2021). Patient generated health data and electronic health record integration in oncologic surgery: a call for artificial intelligence and machine learning. J Surg Oncol.

[106] Coravos A, Doerr M, Goldsack J, Manta C, Shervey M, Woods B, Wood WA (2020). Modernizing and designing evaluation frameworks for connected sensor technologies in medicine. NPJ Digit Med.

[107] Cho S, Ensari I, Weng C, Kahn M, Natarajan K (2021). Factors affecting the quality of person-generated wearable device data and associated challenges: rapid systematic review. JMIR Mhealth Uhealth.

