# Supplementary material for: Recommendations for the Quality Management of Patient-Generated Health Data in Remote Patient Monitoring: Mixed Methods Study
Source: JMIR Mhealth Uhealth. 2023 Feb 24;11:e35917. doi: 10.2196/35917 (PMC10007009; doi:10.2196/35917)
Supplement: Multimedia Appendix 1 [file mhealth_v11i1e35917_app1.docx]

**Multimedia Appendix 1**

This appendix represents seven tables for the seven data quality management aspects of PGHD; each describes the key findings from previous studies of the project and the common themes from the findings for interpretation and construction of the recommendations.

**PGHD accessibility**

Table 1 PGHD accessibility: findings and main themes for interpretation

| **Considerations identified in literature review** | - Patients may conceal some data from clinicians to pretend pursuing the care plans - The sharing PGHD platforms may raise concerns over data access by others - Many self-logging wearables do not have data export features other than APIs that share data to social network websites. This makes it challenging when clinicians need to review these data daily - Lack of PGHD ownership principles to determine who can grant data access to others |
| --- | --- |
| **Challenges identified in interviews** | - Clinicians cannot access new data when patients change CGM - Lack of organisational ethics approach for data access - Lack of IT staff to help clinicians access reports - Lack of cybersecurity protocols to hinder wearables data hacking |
| **Further challenges identified in the workshop** | - Lack of transparency on who owns the data - Lack of consent for continuous data collection and use - Lack of patients’ access to raw data |
| **Solutions and expectations expressed in the workshop** | - Develop data ownership principles - Design notifications in the wearable platform to alert patients once data are accessed by others - Provide dynamic data authorisation - Provide patients’ access to raw data - Define wearable cybersecurity standards - Create data encryption techniques - Consider privacy in the wearable design - Patients expect the wearable developer to provide transparent notice to patients on data governance. - Patients expect that clinicians provide informed consents addressing how patients or carers can access and use PGHD securely and transparently. - Patients want to access their data from the wearable platforms for free. |
| **Main themes for interpretation** | Theme 1: Patients’ and clinicians’ access to PGHD  Theme 2: Patients’ and clinicians’ awareness of PGHD access by others  Theme 3: Patients’ consent to PGHD access by others |

**PGHD accuracy**

Table 2 PGHD accuracy: findings and main themes for interpretation

| **Considerations identified in the literature review** | - Wearable functionality may lead to inaccurate data. Therefore, the devices should be calibrated often to make sure they work correctly and collect accurate data |
| --- | --- |
| **Challenges identified in interviews** | - Sometimes CGM reads higher values than the actual measurement - Patient's illness or dehydration affects the sensor functionality - Clinicians and CGM manufacturers disagree on calibration frequency - Potentials for errors in manual data entry into CGM - Wrong application of wearable on the body - Burden on patients using CGMs |
| **Further challenges identified in the workshop** | - Inaccurate data due to the use of different wearables with different accuracy standard levels - Lack of data editing functionalities |
| **Solutions and expectations expressed in the workshop** | - Define standardised accuracy levels of PGHD - Need for continuous wearable testing - Wearable developers should adopt accuracy-related feedback from patients and clinicians - Enable data edit functionality in the wearable platforms - Clinicians should educate patients on the best practices of PGHD collection and sharing. - Patients should provide continuous feedback about the technical and operational issues to wearable developer companies |
| **Main themes for interpretation** | Theme 1: Automatic and manual PGHD collection  Theme 2: PGHD annotation  Theme 3: Wearable calibration |

**PGHD completeness**

As this DQM aspect was not raised in the initial literature review study, there is no related section for it in Table 3, rather it is supported by several sources from the updated literature review throughout the corresponding section interpretation in the manuscript.

Table 3 PGHD completeness: findings and main themes for interpretation

| **Challenges identified in interviews** | - Incomplete data result in repeating data collection order - Battery problems - Wearable dysfunction - Patients may forget to collect data and provide complete report |
| --- | --- |
| **Further challenges identified in the workshop** | - Lack of access to the Internet to send the collected data - Incompleteness of data entered manually - Lack of data synchronisation due to moving geographically across time zones |
| **Solutions and expectations expressed in the workshop** | - Design notification to alert for missing data - Patients’ education and engagement |
| **Main themes for interpretation** | Theme 1: No active PGHD collection  Theme 2: Resuming PGHD collection after downtime  Theme 3: Context for incomplete PGHD |

**PGHD consistency**

Table 4 PGHD consistency: findings and main themes for interpretation

| **Considerations identified in the literature review** | - Data are not documented and transmitted through standardised formats |
| --- | --- |
| **Challenges identified in interviews** | - It is difficult to normalise data from different wearables into one platform - PGHD collection from different wearable platforms leads to inconsistent reports - Inconsistent data collection from different wearables leads to inconsistent data presentation - Various inconsistent reports that do not talk to each other will not provide a clear picture of the patient's status |
| **Further challenges identified in the workshop** | - Lack of awareness of data flow and data management |
| **Solutions and expectations expressed in the workshop** | - Develop data consistency checking mechanisms to correlate with other data sources - Incorporate data with the clinical workflow - Invest in appropriate IT infrastructure that enables PGHD integration with EMR systems at scale - Patients can discuss their preferred wearables and associated platforms with RPM team to identify ways to share PGHD consistently - Define consistency strategies to incorporate PGHD with other clinical data of patient record - Wearable developers should create devices that comply with the defined data exchange standards in healthcare settings |
| **Main themes for interpretation** | Theme 1: PGHD definitions and formats  Theme 2: PGHD integration with electronic medical records  Theme 3: PGHD exchange within and outside care settings |

**PGHD interpretability**

Table 5 PGHD interpretability: findings and main themes for interpretation

| **Considerations identified in the literature review** | - PGHD are not presented similarly to clinical data. PGHD are captured dynamically, and therefore the patterns are unfamiliar to clinicians. They may make inaccurate decisions and endanger patient’s health - Lack of standardised data presentation formats lead to problems in interpreting PGHD - Poor interfaces may lead to poor patient-clinician communication as they draw clinicians’ attention toward the device or portal rather than the patient - Clinicians are concerned that patients’ focus on wearables data may threaten their autonomy - Static PGHD visualisations do not allow clinicians to further investigate the reports - Lack of clinicians’ training on PGHD lead to difficulty in understanding and interpreting the data |
| --- | --- |
| **Challenges identified in interviews** | - When patients do not understand the represented data, they become less motivated to use the wearable continuously - A large amount of data is displayed in the report which needs cleaning and editing - Report formats of sleep wearable are not user-friendly - Clinicians and wearable developers have different views on report formats - Difficulty in interpreting different reports from different wearables - Lack of contextual data |
| **Further challenges identified in the workshop** | - Lack of contextual data from consumer wearables to supplement medical wearables data to be easily understood |
| **Solutions and expectations expressed in the workshop** | - Collect contextual data and evaluate their quality - Design standardised data presentation formats for clinicians across various wearables used in RPM - Wearables can be developed based on patient-centred care models - Clinicians suggest the involvement of health information professionals to analyse and process PGHD and provide meaningful information for clinicians. |
| **Emerged themes for interpretation** | Theme 1: PGHD contextualisation  Theme 2: Dynamic and static PGHD visualisation  Theme 3: The patient’s understanding of PGHD |

**PGHD relevancy**

Table 6 PGHD relevancy: findings and main themes for interpretation

| **Considerations identified in the literature review** | - Patients’ reasons for collecting PGHD are influenced by their knowledge and understanding of their health condition. However, these data may not be relevant from the clinician’s perspective - Identifying relevant, pertinent lifestyle data is challenging - The usefulness of PGHD in clinic visits relies on the clinical relevance of these data. Whereas most of the data are not yet proven to be relevant for use in patient care |
| --- | --- |
| **Challenges identified in interviews** | - Clinicians spend much time on prioritising the most relevant information from the large amount of data presented in the reports - Different departments have different criteria for what information are relevant for presentation on the report |
| **Further challenges identified in the workshop** | - Different clinical judgement on data relevancy |
| **Solutions and expectations expressed in the workshop** | - Improve health literacy to understand the relevance of data to the standards of care - Ensure shared understanding of data relevancy among patients and clinicians - Design wearables exclusively for the intended purpose of use and align with the standards of care in the healthcare settings |
| **Main themes for interpretation** | Theme 1: PGHD relevancy to the standards of care |

**PGHD timeliness**

Table 7 PGHD timeliness: findings and main themes for interpretation

| **Considerations identified in the literature review** | - Lack of clinicians’ access to PGHD in real-time during data collection |
| --- | --- |
| **Challenges identified in interviews** | - Data are not transmitted to clinicians on a real-time basis - Data are accessible to clinicians in different timelines in public care systems where patients see different clinicians - Lack of protocols for timely data sharing and retrospective data access reduces the clinical value of PGHD |
| **Further challenges identified in the workshop** | - High volume of unfiltered data to be timely - Lack of consensus among PGHD stakeholders about the definition of timeliness depending on the patient’s status (stable/unstable, at risk, etc.) - Lack of alert systems to patients on the time of data availability |
| **Solutions and expectations expressed in the workshop** | - Automation and AI; to accelerate data filtering so that important data can be available in a timely manner - Enable patients to take responsibility in deciding when health issues need to be escalated - Design alerts for critical indicators to patients and clinicians |
| **Main themes for interpretation** | Theme 1: PGHD availability to patient when needed  Theme 2: PGHD availability to clinician when needed  Theme 3: Timeframe for PGHD sharing between patient and clinician |
